# Effect of Nitrogen-Containing Regulators on Graphene Quantum Dot/ZIF-8 Composites for Photocatalytic CO_2_ Reduction

**DOI:** 10.3390/nano16181126

**Published:** 2026-09-08

**Authors:** Lei Wang, Xinyuan Gao, Shang Li, Shuangyan Li, Weitao Li

**Affiliations:** College of Materials Electronics and Energy Storage, Zhongyuan University of Technology, Zhengzhou 450007, China; wanglei@zut.edu.cn (L.W.); gaoxinyuan408@163.com (X.G.); lishang@zut.edu.cn (S.L.); lishuangyan@zut.edu.cn (S.L.)

**Keywords:** graphene quantum dots, ZIF-8, nitrogen-containing regulator, photocatalytic CO_2_ reduction, photoelectrochemical response

## Abstract

Graphene quantum dots (GQDs) with distinct optical responses were prepared from pyrene using urea, melamine, and 2,4-pyridinedicarboxylic acid as nitrogen-containing regulators and were subsequently combined with ZIF-8 for visible-light-driven CO_2_ reduction. FT-IR, Raman, XRD, optical spectroscopy, representative high-magnification transmission electron microscopy, and X-ray photoelectron spectroscopy (XPS) showed regulator-dependent structural, compositional, and optical differences. XPS detected surface nitrogen in all three GQD samples, with the highest N content in y-GQDs, while the relative N 1s component distributions differed across the series. At a nominal 4 wt% GQD addition, r-GQDs/ZIF-8 gave the highest observed mean CO and CH_4_ formation rates of 23.51 ± 0.48 and 4.08 ± 0.15 μmol·g^−1^·h^−1^, respectively, corresponding to approximately 2.9- and 4.5-fold increases over pristine ZIF-8. This sample also showed the lowest fitted charge-transfer resistance, the highest mean photocurrent density, and the fastest qualitative time-resolved photoluminescence decay among the compared composites. Across three independent five-cycle tests, 83.78 ± 0.54% of the initial combined CO and CH_4_ rate was retained. These results establish correlations among regulator identity, surface composition, optical relaxation, photoelectrochemical response, and catalytic activity, but do not determine a unique charge-transfer pathway or exclude contributions from surface basicity, CO_2_ adsorption, and nominal-loading differences.

## 1. Introduction

The continuous increase in atmospheric carbon dioxide concentration associated with fossil-fuel consumption has intensified environmental and energy-related challenges. Photocatalytic conversion of CO_2_ into carbon-containing products offers a potential route for coupling carbon utilization with solar-energy storage [1,2,3]. Nevertheless, photocatalytic CO_2_ reduction is commonly limited by insufficient visible-light absorption, rapid recombination of photogenerated charge carriers, sluggish interfacial electron transfer, and the thermodynamic stability of CO_2_ [1,2]. Developing photocatalysts that combine light harvesting, charge transport, and accessible reaction sites therefore remains important.

Metal–organic frameworks (MOFs) have attracted attention as photocatalytic platforms because of their high surface areas, tunable pore structures, and adjustable coordination environments [4,5]. ZIF-8, a representative zeolitic imidazolate framework, possesses a stable porous architecture and channels that can support molecular transport [4,5,6,7]. However, pristine ZIF-8 absorbs mainly in the ultraviolet region and generally shows limited visible-light photoactivity [6,7]. Coupling ZIF-8 with light-responsive nanomaterials has therefore been investigated as a strategy for modifying its optical and photoelectrochemical behavior [8,9,10].

Graphene quantum dots (GQDs) are zero-dimensional carbon nanomaterials with size-dependent electronic structures, abundant edge sites, good dispersibility, and tunable surface functionality [11,12,13,14]. In photocatalytic systems, GQDs have been investigated as light-responsive components, electron mediators, or interfacial modifiers [3,11,15,16]. Their optical response and surface states can vary with precursor selection and heteroatom-containing synthesis reagents [17,18,19,20,21,22]. Nitrogen-containing reagents may therefore influence the carbonization products, surface composition, and emissive states. In the present work, X-ray photoelectron spectroscopy (XPS) was used to compare the surface nitrogen content and N 1s component distributions of the three regulator-derived samples, while the assignments were interpreted as surface-sensitive evidence rather than a complete bulk compositional analysis. More broadly, theoretical work on codoped perovskite photocatalysts demonstrates that deliberate compositional regulation can alter electronic structure and photoresponse for solar-energy conversion [23]. Although that study concerns H_2_ evolution rather than CO_2_ reduction, it provides complementary materials-design context for the regulator-dependent optical trends examined here.

Urea, melamine, and 2,4-pyridinedicarboxylic acid were selected as chemically distinct regulators: a small carbonyl-containing nitrogen source, an N-rich triazine, and an aromatic N-heterocycle bearing carboxyl groups, respectively. Under otherwise identical hydrothermal conditions, this set provides a comparative means of examining how regulator chemistry relates to the surface composition and optical properties of the resulting carbonaceous dots. Previous studies have shown that nitrogen-containing precursors can affect the structure and optical response of GQDs [17,18,19,20,21,22], but direct comparisons of these three regulators within the same GQD/ZIF-8 preparation and photocatalytic protocol remain limited. The XPS comparison was therefore used to test whether the regulator choice was associated with measurable surface-compositional differences, without assuming that precursor identity maps uniquely onto one nitrogen configuration.

In this work, three samples were synthesized from pyrene using urea, melamine, and 2,4-pyridinedicarboxylic acid and were denoted b-GQDs, y-GQDs, and r-GQDs, respectively. They were combined with ZIF-8 using nominal addition amounts of 1–5 wt%. The working hypothesis was that chemically distinct regulators would yield products with different surface compositions and optical responses and that these differences would correlate with the photoelectrochemical behavior and CO_2_ reduction performance of the corresponding composites. Structural and spectroscopic results, XPS, representative high-magnification transmission electron microscopy (TEM), relative fluorescence quantum yields, time-resolved photoluminescence (PL), electrochemical impedance spectroscopy (EIS), transient photocurrent responses, photocatalytic rates, and five-cycle stability were compared across the sample series. This comparative design evaluates observable correlations without presupposing a unique nitrogen configuration or interfacial electron-transfer pathway.

## 2. Materials and Methods

### 2.1. Materials

Pyrene, urea, melamine, 2,4-pyridinedicarboxylic acid, zinc nitrate hexahydrate (Zn(NO_3_)_2_·6H_2_O), 2-methylimidazole, methanol, ethanol, Nafion solution, sodium sulfate, and quinine sulfate were used in this study. All reagents were of analytical grade and were used as received without further purification. Deionized water was used throughout the experiments.

### 2.2. Synthesis of Nitrogen-Source-Regulated GQDs

Nitrogen-containing-regulator-derived GQD samples were synthesized through a one-step hydrothermal route using pyrene as the carbon precursor and urea, melamine, or 2,4-pyridinedicarboxylic acid as the regulator. Typically, 0.01 g of pyrene and 0.01 g of the selected regulator were dispersed in 10 mL of ethanol and ultrasonicated for 10 min. The suspension was transferred to a Teflon-lined stainless-steel autoclave and heated at 180 °C for 12 h. After natural cooling to room temperature, the reaction mixture was centrifuged to remove insoluble residues and large particles. The clarified supernatant was collected, and centrifugation was repeated until no visible suspended particles remained. No dialysis or ultrafiltration step was performed. The clarified solution was dried at 60 °C to obtain solid products. Samples prepared using urea, melamine, and 2,4-pyridinedicarboxylic acid were denoted b-GQDs, y-GQDs, and r-GQDs, respectively, according to their blue, yellow, and red emission.

### 2.3. Synthesis of ZIF-8

ZIF-8 was synthesized by a room-temperature solution precipitation method. Zn(NO_3_)_2_·6H_2_O (1.5 g) was dissolved in 70 mL of methanol under magnetic stirring to form a clear zinc precursor solution. Separately, 3.3 g of 2-methylimidazole was dissolved in 70 mL of methanol to prepare the ligand solution. The zinc precursor solution was slowly added to the ligand solution under continuous stirring, and the resulting mixture was stirred at room temperature for 24 h. The white precipitate was collected by centrifugation at 5000 rpm for 5 min and washed three times with 50 mL of methanol to remove residual ligands and unreacted zinc salts. The product was dried in a vacuum oven at 80 °C for 6 h to obtain ZIF-8 powder.

### 2.4. Preparation of GQDs/ZIF-8 Composites

GQDs/ZIF-8 composites were prepared using a solution-assisted surface-loading method. ZIF-8 powder (100 mg) was dispersed in 20 mL of methanol and ultrasonicated for 15 min. The required amount of b-GQD, y-GQD, or r-GQD dispersion was then slowly added under continuous magnetic stirring. The GQD addition amounts were set to 1, 2, 3, 4, and 5 wt% relative to the initial mass of ZIF-8. The mixtures were stirred at room temperature for 4 h. The solids were collected by centrifugation at 5000 rpm for 5 min, washed several times with methanol to remove weakly adsorbed material, and dried at 80 °C for 6 h. The composites are denoted x wt% b-GQDs/ZIF-8, x wt% y-GQDs/ZIF-8, and x wt% r-GQDs/ZIF-8, where x represents the nominal GQD addition. Because the retained GQD content after washing was not independently quantified, these labels do not represent measured final compositions. Accordingly, the wt% values are operational input ratios rather than analytically determined final compositions. Any apparent optimum across the loading series must therefore be interpreted as a nominal-loading trend and not as a concentration-dependent structure–property relationship.

### 2.5. Characterization

The crystalline structures of the samples were examined by X-ray diffraction (XRD) on a D2 PHASER diffractometer (Bruker, Karlsruhe, Germany) with Cu Kα radiation. Fourier transform infrared (FT-IR) spectra were collected using an ALPHA II spectrometer (Bruker, Ettlingen, Germany) with the KBr pellet method over 4000–400 cm−1. Raman spectra were recorded in point-scan and mapping modes using an XploRA PLUS Raman spectrometer (HORIBA, Longjumeau, France) with a 532 nm excitation laser. The morphologies of pristine ZIF-8 and the selected composite were observed using a field-emission scanning electron microscope (ZEISS Sigma 360, Carl Zeiss, Jena, Germany). Representative high-magnification transmission electron micrographs of the regulator-derived GQD samples were acquired at an accelerating voltage of 200 kV. Ultraviolet–visible (UV–vis) absorption spectra were recorded on a LAMBDA 1050+ UV–vis–NIR spectrophotometer (PerkinElmer, Shelton, CT, USA) over 200–800 nm. PL and PL excitation spectra were measured using an RF-6000 fluorescence spectrophotometer (Shimadzu, Kyoto, Japan). Relative fluorescence quantum yields were determined using quinine sulfate in 0.1 M H2SO4 as the reference (Φ = 58%). Normalized time-resolved PL decays of the isolated GQDs and corresponding nominal 4 wt% composites were recorded over 0–60 ns and compared qualitatively without assigning a unique exponential model. X-ray photoelectron spectroscopy (XPS) was performed using a spectrometer equipped with a monochromatic Al Kα X-ray source to evaluate the surface elemental composition and the C 1s, N 1s, O 1s, and Zn 2p regions. Binding energies were referenced to the C 1s peak at 284.8 eV; small shifts were interpreted cautiously and were not used alone to establish the direction of charge transfer. Thermogravimetric curves were obtained using a TG 209 F1 analyzer (NETZSCH, Selb, Germany) to compare the thermal decomposition profiles of ZIF-8 and the composites.

### 2.6. Photoelectrochemical Measurements

Photoelectrochemical measurements were performed on a CHI600E electrochemical workstation (Shanghai Chenhua Instrument Co., Shanghai, China) using a three-electrode configuration. Catalyst-coated fluorine-doped tin oxide (FTO) glass, Pt foil, and an Ag/AgCl electrode were used as the working, counter, and reference electrodes, respectively. To prepare the working electrode, 5 mg of photocatalyst was dispersed in 950 μL of an ethanol/water mixture and 50 μL of Nafion solution, followed by ultrasonication for 30 min. The ink was drop-cast onto the same defined FTO area for each sample and dried at 60 °C. A 0.5 M Na_2_SO_4_ aqueous solution was used as the electrolyte. Transient photocurrent responses were recorded under a common fixed applied bias and intermittent visible-light irradiation from a 300 W Xe lamp (PLS-SXE300, Perfect Light, Beijing, China) equipped with a 420 nm cutoff filter; the light-on and light-off intervals were each 30 s. The current was normalized to the illuminated electrode area. EIS data were recorded over 100 kHz–0.1 Hz using a 5 mV AC perturbation under the same series-level measurement conditions. Nyquist data were fitted using an R_s_-(R_ct_||CPE) equivalent circuit. Three independently prepared electrodes were measured for each sample, and fitted R_ct_ values and photocurrent densities are reported as mean ± standard deviation (SD, *n* = 3). The absolute DC potential was not retained in the exported acquisition table; therefore, the photoelectrochemical data are interpreted only as within-series comparisons and are not compared quantitatively with literature values obtained under different electrochemical conditions.

### 2.7. Photocatalytic CO_2_ Reduction Tests

Photocatalytic CO_2_ reduction experiments were performed in a sealed 100 mL quartz reactor under visible-light irradiation. In a typical experiment, 10 mg of photocatalyst was dispersed in 10 mL of deionized water by ultrasonication for 15 min and then transferred to the reactor. Before irradiation, high-purity CO_2_ was introduced continuously for 30 min to remove residual air and establish a CO_2_-containing atmosphere. A 300 W Xe lamp (PLS-SXE300, Perfect Light, China) equipped with a 420 nm cutoff filter was used as the light source. The suspension was stirred continuously, and circulating cooling water was used to limit heating. The evolved gases were automatically sampled every 30 min and analyzed using a GC-2060 gas chromatograph (Shimadzu, Kyoto, Japan) equipped with a thermal conductivity detector (TCD). CO and CH_4_ were assigned by retention time and quantified using external calibration curves. The formation rate of product i was calculated as r_i_ = n_i_/(m × t), where n_i_ is the amount of product detected, m is the catalyst mass, and t is the irradiation time. Product formation rates are reported in μmol·g^−1^·h^−1^. Each activity measurement was repeated in three independent photocatalytic runs, and the results are reported as mean ± SD (*n* = 3). The loading-series differences are discussed descriptively, and no statistical significance is inferred. Control tests for nominal 4 wt% r-GQDs/ZIF-8 were conducted under otherwise identical conditions in the dark, without catalyst, and under N_2_ instead of CO_2_. Reusability was evaluated through three independent five-cycle tests, and retention was calculated from the combined CO and CH_4_ formation rate relative to the first cycle of each run. The apparent quantum efficiency (AQE) measurements were carried out under the same photocatalytic CO_2_ reduction conditions, except that band-pass filters with different center wavelengths were used to obtain monochromatic irradiation. A PL-MW2000 optical power meter was used to measure the incident light intensity at the catalyst surface. Band-pass filters centered at 365, 420, 500, 550, and 600 nm with a half-width of ±10 nm were used. The AQE values were calculated from the amount of generated CO and the number of incident photons.

## 3. Results and Discussion

### 3.1. Representative TEM and Local Lattice Features of Regulator-Derived GQDs

**Figure 1:**
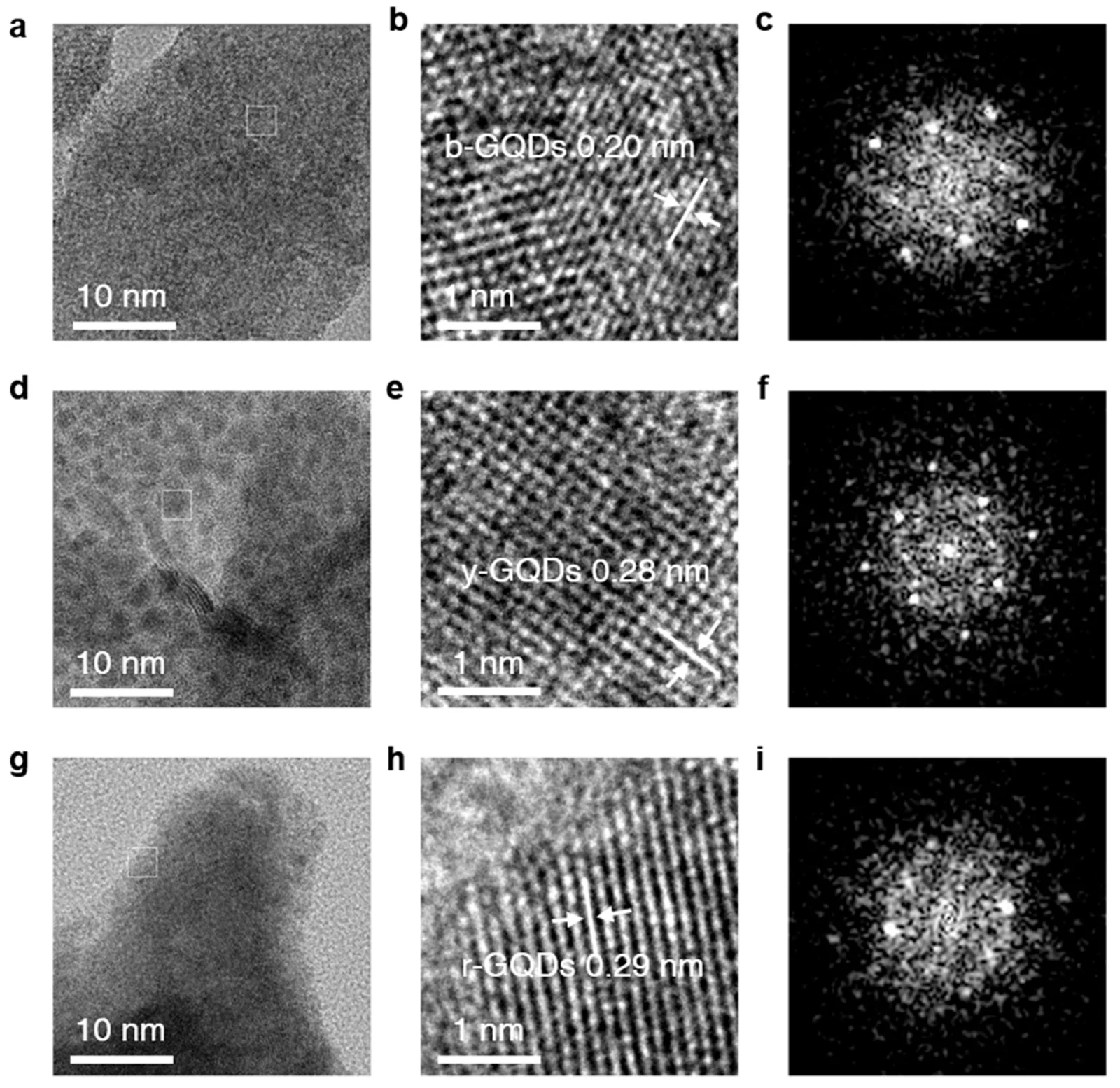
Representative electron-microscopy characterization of regulator-derived GQDs. TEM images of (**a**) b-GQDs, (**d**) y-GQDs, and (**g**) r-GQDs; corresponding HRTEM images (**b**,**e**,**h**) with the annotated local lattice spacings; and FFT patterns (**c**,**f**,**i**). The micrographs are representative local views and were not used to derive an average particle size or particle-size distribution; the FFT patterns are not treated as substitutes for selected-area electron diffraction.

Representative TEM images of b-GQDs, y-GQDs, and r-GQDs are shown in Figure 1a,d,g. The selected fields contain nanoscale carbonaceous domains, while the corresponding high-resolution transmission electron microscopy (HRTEM) images in Figure 1b,e,h show local lattice fringes. The annotated local spacings are approximately 0.20, 0.28, and 0.29 nm for the b-, y-, and r-derived samples, respectively.

The corresponding fast Fourier transform (FFT) patterns are shown in Figure 1c,f,i. These patterns are consistent with periodic contrast within the selected local regions, but they are not treated as substitutes for selected-area electron diffraction and are not used to establish a bulk crystalline phase or a unique crystallographic orientation.

Because only a limited number of high-magnification fields were available, the images provide representative local structural evidence rather than statistically sufficient morphology. No average particle diameter or particle-size distribution is therefore derived from Figure 1. A defensible particle-size distribution would require low-magnification images containing a sufficiently large, randomly selected particle population and a defined counting protocol; these data were not available for the present revision.

### 3.2. Optical Properties of Nitrogen-Source-Regulated GQDs

**Figure 2:**
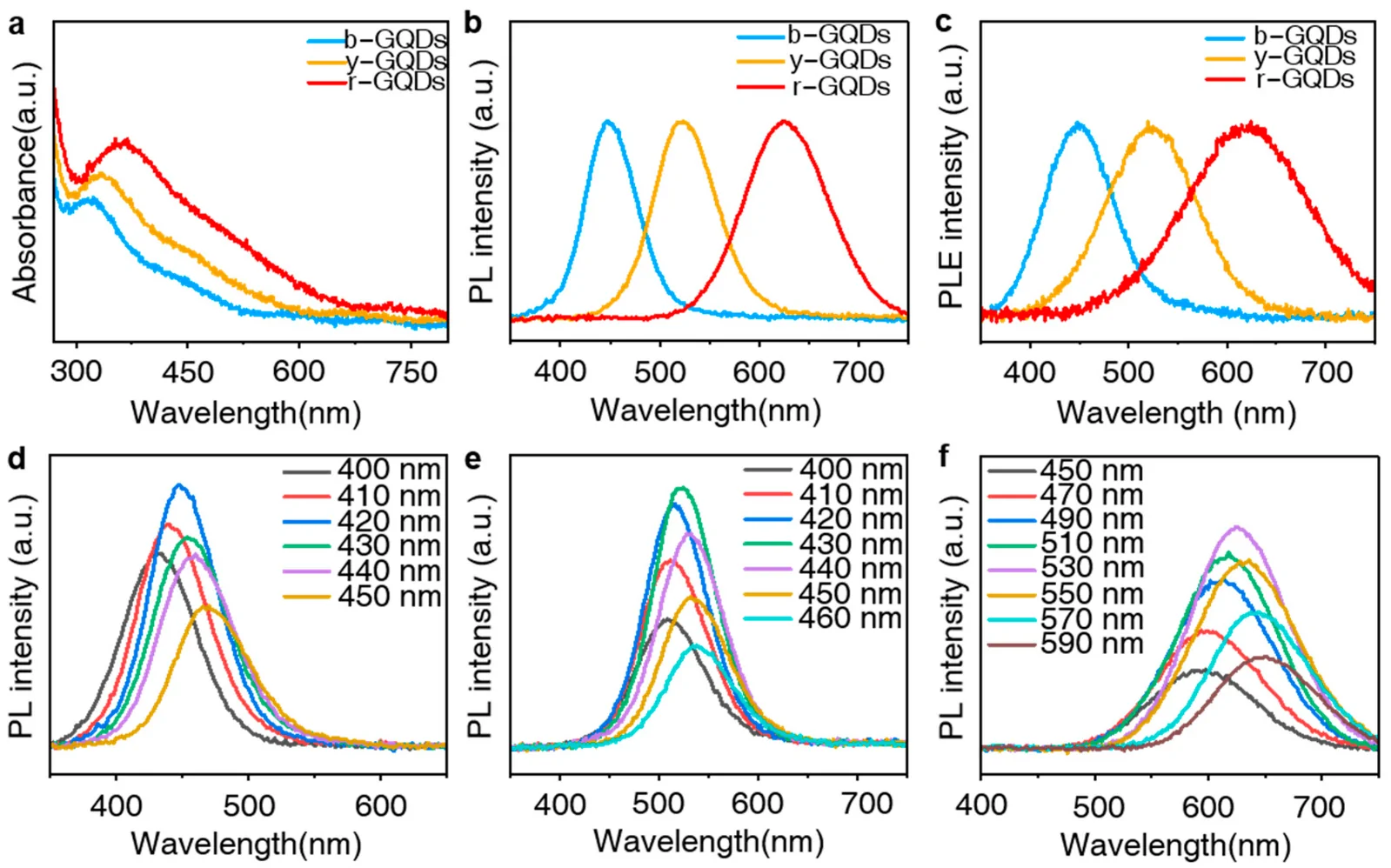
Optical properties of regulator-derived GQDs: (**a**) UV–vis absorption spectra, (**b**) representative PL spectra, (**c**) PLE spectra, and excitation-dependent PL spectra of (**d**) b-GQDs, (**e**) y-GQDs, and (**f**) r-GQDs. The relative quantum-yield values are reported in the text and Table S4. Because intensity is reported in arbitrary units, each excitation-dependent panel should be interpreted for its within-panel spectral evolution; no quantitative comparison of absolute intensity across panels (**d**–**f**) is intended.

The optical properties of the GQD samples were evaluated using UV–vis absorption, PL, PL excitation (PLE), excitation-dependent PL, and relative fluorescence quantum-yield measurements. All three samples exhibited strong ultraviolet absorption and absorption tails extending into the visible region (Figure 2a). These features can include contributions from π–π* transitions of sp^2^ carbon domains and lower-energy transitions associated with surface or defect-related states [24,25]. The differences among the absorption profiles show that the regulator used during synthesis was associated with different optical responses, but they do not establish a specific electronic structure. The corresponding maximum relative quantum yields are summarized in Table S4.

The PL spectra in Figure 2b showed emission maxima at approximately 448, 524, and 624 nm for b-GQDs, y-GQDs, and r-GQDs, respectively. The red shift across the series may reflect differences in conjugated domains, surface functionality, and emissive states [11,19,22,26,27]. The corresponding PLE spectra also differed among the samples (Figure 2c), supporting regulator-dependent excitation and emission behavior without uniquely assigning the underlying states. In chemical terms, the three regulators differ in nitrogen content, aromaticity, and oxygen-containing functionality; these differences can change the relative contributions of sp^2^ carbon-domain states and edge/surface-associated states during carbonization. The observed higher surface N content for y-GQDs, higher surface O content for r-GQDs, and red-shifted r-GQD emission are consistent with a changed distribution of these states, which can alter excitation-energy relaxation. However, XPS and steady-state optical spectra do not by themselves resolve the molecular identity or relative population of each emissive state.

The excitation-dependent spectra in Figure 2d–f further revealed regulator-dependent emission behavior. In particular, r-GQDs emitted mainly in the 594–652 nm region and showed changes in intensity and peak position with excitation wavelength. Such excitation dependence is consistent with multiple emissive contributions from carbon-domain and surface-associated states [24,26,27], although their chemical identities were not resolved.

Using quinine sulfate as the reference, the maximum relative fluorescence quantum yields reported for b-GQDs, y-GQDs, and r-GQDs were 38.35%, 32.13%, and 20.09%, respectively. These values indicate different overall balances between radiative and non-radiative relaxation in the isolated GQD samples. The order was opposite to the photocatalytic activity of the corresponding GQDs/ZIF-8 composites, showing that isolated-GQD quantum yield alone is not a sufficient performance descriptor. Time-resolved PL measurements performed directly on the composites are considered in Section 3.4. For the composites, a high fluorescence quantum yield of the isolated GQD is not necessarily beneficial: it indicates efficient radiative relaxation in the isolated dispersion, whereas productive photocatalysis additionally requires charge generation, interfacial separation, and surface reaction. The inverse quantum-yield/activity ordering, together with the faster qualitative composite PL decay and lower fitted impedance for r-GQDs/ZIF-8, is therefore consistent with greater competition from non-radiative or interfacial pathways in the composite, but it does not identify the direction or microscopic mechanism of electron transfer.

### 3.3. Structural and Surface-Chemical Characteristics of Regulator-Derived GQDs

**Figure 3:**
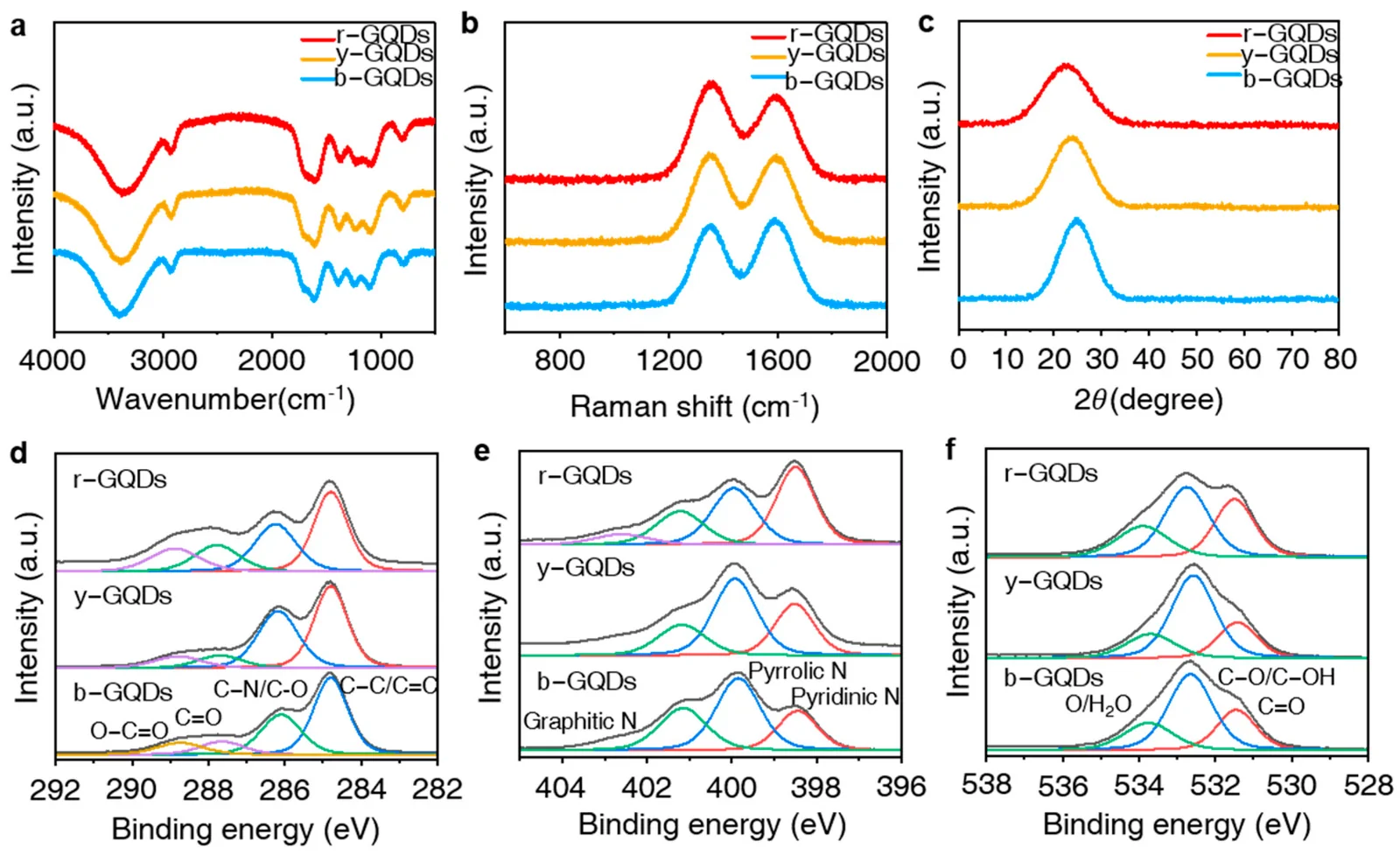
Structural and XPS characterization of regulator-derived GQDs: (**a**) FT-IR spectra, (**b**) Raman spectra, and (**c**) XRD patterns; high-resolution (**d**) C 1s, (**e**) N 1s, and (**f**) O 1s spectra arranged from top to bottom as r-GQDs, y-GQDs, and b-GQDs. In panels (**d**–**f**), the black/grey curves represent the measured XPS spectra, the red curves represent the overall fitted envelopes, and the colored component curves represent the deconvoluted chemical-state contributions labeled in each panel. Surface compositions and N 1s fit parameters are summarized in Tables S1 and S2.

The FT-IR spectra in Figure 3a show broad bands at 3200–3500 cm^−1^ that are consistent with O–H and/or N–H stretching vibrations [17,18]. Bands near 1600 cm^−1^ can include contributions from C=C and C=N vibrations, while signals within 1000–1500 cm^−1^ are consistent with C–N, C–O, and related functionalities. Differences in peak shape and relative intensity indicate regulator-dependent spectral differences [17,18]. FT-IR alone cannot quantify nitrogen content or establish a specific bonding configuration.

The Raman spectra in Figure 3b contain the characteristic D and G bands of carbon materials at approximately 1350–1400 and 1580–1600 cm^−1^, respectively [19,20,21,28]. The presence of both bands is consistent with disordered graphitic domains, and the intensity ratio I_G_/I_D_ was 0.91. The broad XRD features in Figure 3c likewise indicate limited long-range order; crystallite size and stacking parameters were not extracted. The broad, partially overlapping carbon-related features and the absence of a validated peak-shape/deconvolution protocol prevented reliable extraction of these parameters. Reporting such values from these patterns would imply a precision not supported by the data.

The XPS survey spectra in Figure S1a contained C, N, and O signals for all three regulator-derived samples. The measured surface atomic percentages of C/N/O were 75.6/9.2/15.2 for b-GQDs, 69.8/16.4/13.8 for y-GQDs, and 67.4/8.7/23.9 for r-GQDs (Table S1). Thus, y-GQDs showed the highest surface-sensitive nitrogen content, whereas r-GQDs showed the highest oxygen content; these values are not interpreted as indicating bulk elemental composition.

The high-resolution C 1s, N 1s, and O 1s profiles are compared in Figure 3d–f. The N 1s profiles contain components near 398.5, 399.9, 401.2, and 402.6 eV, assigned to pyridinic-, pyrrolic/amine-, graphitic-, and oxidized-N environments, respectively (Figure 3e and Table S2). Differences in the component distributions support regulator-dependent surface chemistry without implying that precursor identity produces one exclusive nitrogen configuration.

### 3.4. Composite Structure, Thermal Behavior, and Charge-Carrier-Related Measurements

**Figure 4:**
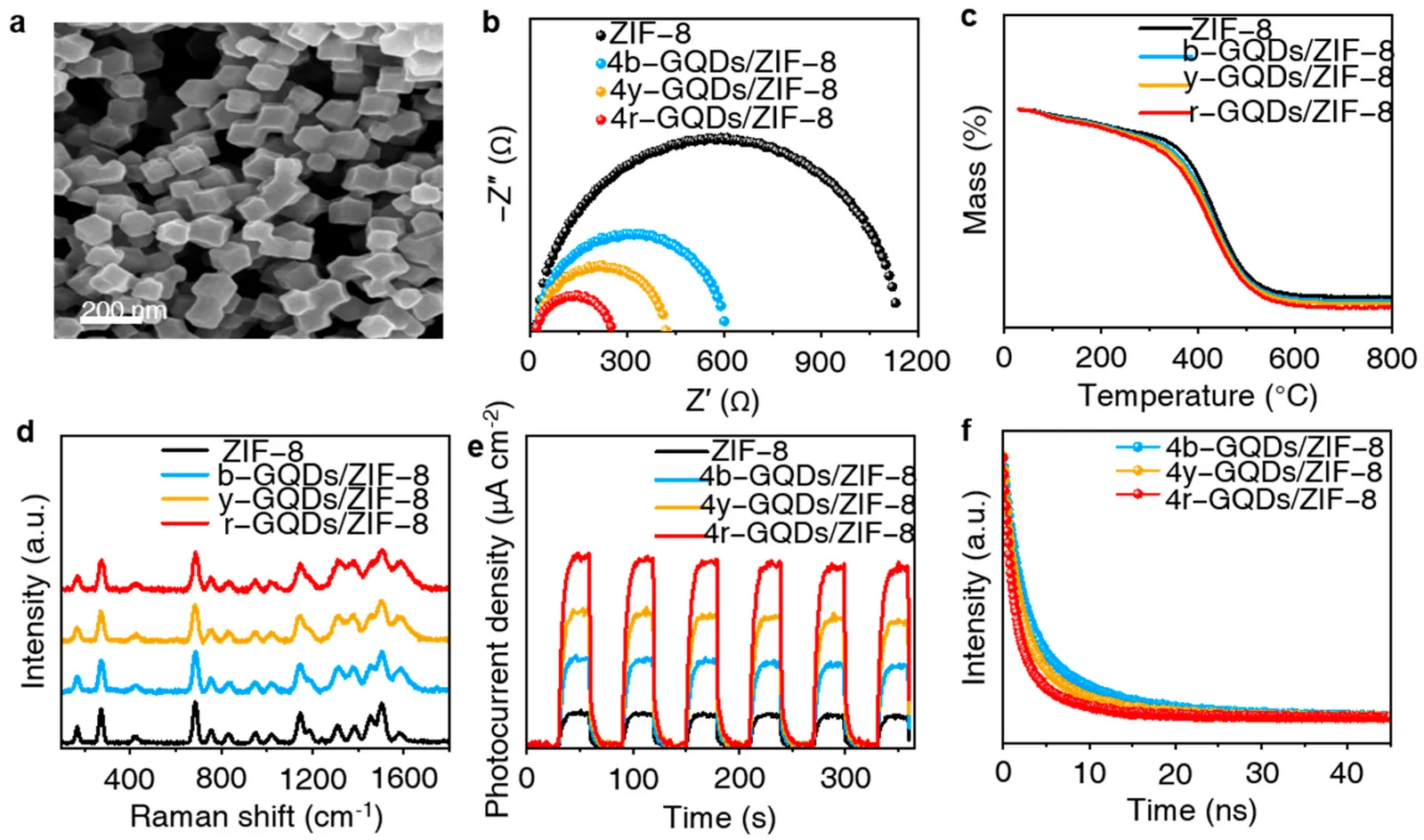
Structural, thermal, photoelectrochemical, and time-resolved PL characterization of ZIF-8 and GQDs/ZIF-8 composites: (**a**) SEM image of pristine ZIF-8; (**b**) representative EIS Nyquist plots; (**c**) TG curves; (**d**) Raman spectra; (**e**) representative transient photocurrent responses; and (**f**) normalized time-resolved PL decays of nominal 4 wt% composites. Replicate photoelectrochemical summaries are provided in Tables S9 and S10. The SEM image is not used to infer individual GQD size or location. For panel (**b**), EIS was recorded from 100 kHz to 0.1 Hz using a 5 mV AC perturbation; for panel (**e**), the electrolyte was 0.5 M Na_2_SO_4_ and the light-on/light-off intervals were 30 s under a 300 W Xe lamp with a 420 nm cutoff filter. The traces in panels (**b**,**e**) are representative; the fitted R_ct_ values and photocurrent densities are reported as mean ± SD from three independently prepared electrodes (*n* = 3) in Tables S9 and S10.

ZIF-8 was used as the host for preparing the GQDs/ZIF-8 composites. Figure 4a shows the morphology of pristine ZIF-8. Because individual GQDs are not resolved in this SEM image, it is not used to infer GQD particle size, dispersion, or location within the composite.

The Raman spectra in Figure 4d retain the principal features of ZIF-8 after composite preparation. Weak carbon-related contributions may overlap with the framework bands, but the spectra do not permit quantitative determination of GQD content or a specific bonding interaction. The complementary FT-IR spectra and XRD patterns are provided in Figure S1b,c. The principal FT-IR band positions and qualitative spectral differences are summarized in Table S7. The principal ZIF-8 diffraction reflections and characteristic imidazolate/Zn–N vibrational features remain observable, while small intensity or position differences are interpreted qualitatively and are not taken as proof of a new interfacial bond [29].

EIS was used to compare the impedance responses of pristine ZIF-8 and the composites. As shown in Figure 4b, pristine ZIF-8 exhibited the largest Nyquist arc, whereas r-GQDs/ZIF-8 exhibited the smallest among the measured samples. Equivalent-circuit fitting yielded mean R_ct_ values of 807.7 ± 30.7, 542.7 ± 19.6, 369.7 ± 17.0, and 235.0 ± 12.8 Ω for ZIF-8, b-GQDs/ZIF-8, y-GQDs/ZIF-8, and r-GQDs/ZIF-8, respectively (mean ± SD, *n* = 3; Tables S9 and S10). The decreasing R_ct_ trend is consistent with lower apparent interfacial impedance within this series [30,31,32,33], but it does not establish a specific charge-transfer pathway.

Thermogravimetric analysis was used to compare the thermal behavior of ZIF-8 and the composites (Figure 4c). Initial mass loss is consistent with the removal of physically adsorbed water and residual solvent, whereas the major loss at higher temperature is associated with decomposition of the organic framework. Because the actual retained GQD contents were not independently measured and the curves overlap closely, the modest residual-mass differences are not used to quantify GQD loading [34,35].

The transient photocurrent responses in Figure 4e show reproducible light-on/light-off switching. Mean photocurrent densities from three independently prepared electrodes were 1.847 ± 0.070, 3.440 ± 0.098, 4.750 ± 0.118, and 6.333 ± 0.127 μA·cm^−2^ for ZIF-8, b-GQDs/ZIF-8, y-GQDs/ZIF-8, and r-GQDs/ZIF-8, respectively (mean ± SD, *n* = 3; Table S10). The higher response of r-GQDs/ZIF-8 is consistent with more effective generation and/or transport of photoinduced charge in this electrode configuration [30,32,33,36], but it does not determine carrier-transfer direction. Because the absolute applied potential was not retained in the exported acquisition table, the values are used only for within-series comparison. The missing acquisition metadata also prevents conversion of the applied bias to a rigorously documented reference scale; consequently, the measurements are not used to claim an absolute photoelectrochemical performance advantage over literature reports.

Normalized time-resolved PL decays measured directly for the nominal 4 wt% composites are shown in Figure 4f. The decay profiles followed the qualitative order b-GQDs/ZIF-8 > y-GQDs/ZIF-8 > r-GQDs/ZIF-8 at longer delay times, with the r-derived composite exhibiting the fastest decay. A faster decay can include multiple radiative and non-radiative pathways; in the absence of an instrument-response function and validated exponential fits, no numerical lifetime or uniquely productive interfacial-transfer rate is assigned.

### 3.5. Photocatalytic CO_2_ Reduction Activity and Cycling Stability

**Figure 5:**
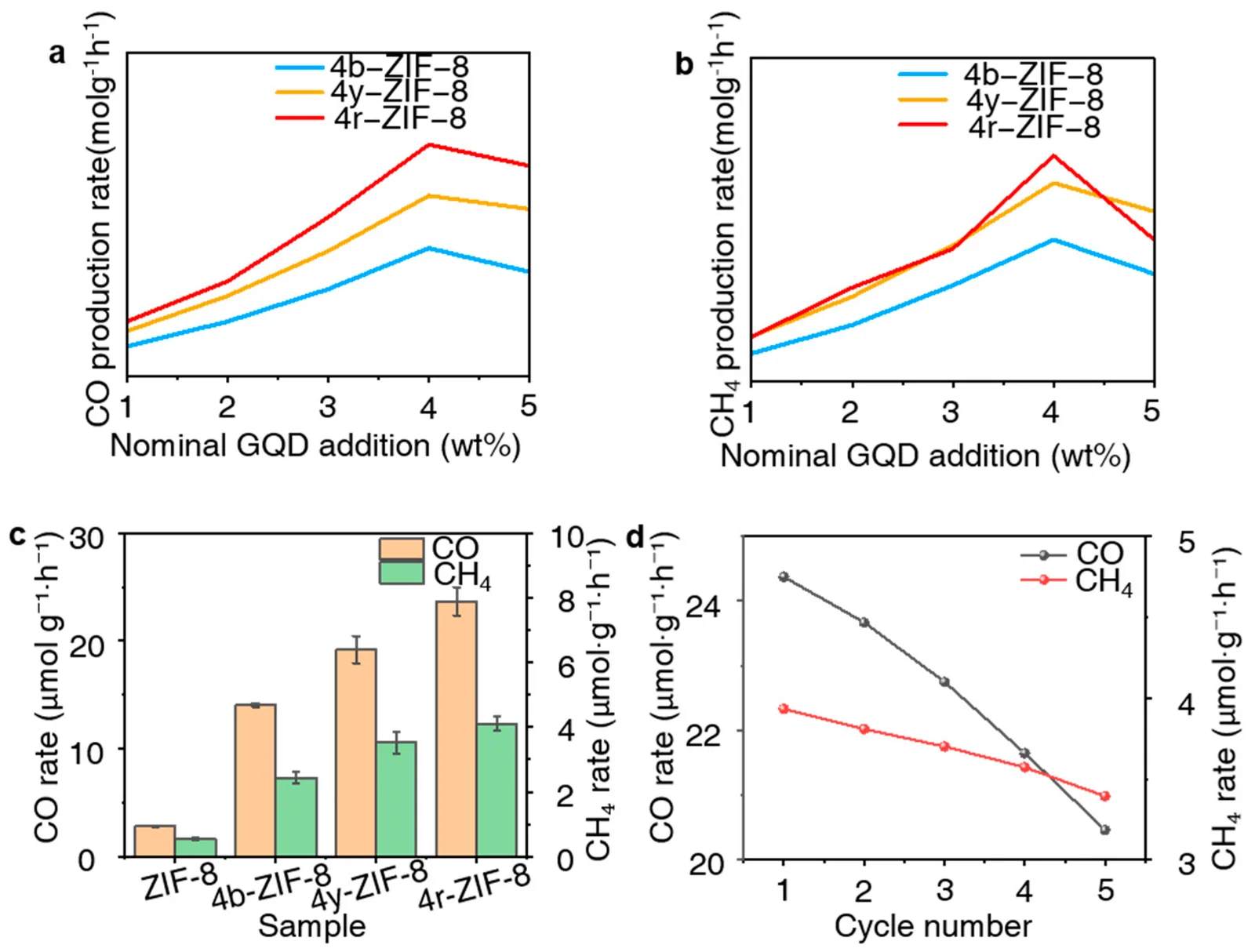
Photocatalytic CO_2_ reduction activity and cycling stability: mean (**a**) CO and (**b**) CH_4_ formation rates as a function of nominal GQD addition; (**c**) mean CO and CH_4_ formation rates for ZIF-8 and the three nominal 4 wt% composites; and (**d**) five-cycle CO and CH_4_ formation rates of nominal 4 wt% r-GQDs/ZIF-8. Error bars in panel (**c**) represent SD from three independent runs (*n* = 3); the *x*-axis of panel (**d**) represents cycle number. Panels (**a**–**c**) were measured in a sealed 100 mL quartz reactor using 10 mg catalyst dispersed in 10 mL water after 30 min CO_2_ purging, under a 300 W Xe lamp with a 420 nm cutoff filter. Panels (**a**,**b**) show mean rates by nominal addition; the individual replicate values, means, and SDs (*n* = 3) are supplied in Table S5. Panel (**d**) summarizes three independent five-cycle tests; its values are mean ± SD (*n* = 3).

The photocatalytic CO_2_ reduction activities of pristine ZIF-8 and the GQDs/ZIF-8 composites were evaluated under visible-light irradiation. As shown in Figure 5a,b and Table S5, the mean CO and CH_4_ formation rates generally increased with nominal GQD addition and approached a broad maximum or plateau at 3–5 wt%, depending on the GQD type and product. The b-derived series showed nearly identical combined mean rates at 3 and 4 wt%, while the y- and r-derived series showed only small differences between 4 and 5 wt% relative to the experimental variation. Among all tested samples, nominal 4 wt% r-GQDs/ZIF-8 gave the highest observed mean CO and CH_4_ rates. This trend is described in terms of nominal GQD addition rather than measured final composition. The amount retained after washing, aggregation, optical shielding, interfacial contact, surface accessibility, and mass transport could all contribute to the loading response [37,38]; the present data do not distinguish among these possibilities.

At a nominal 4 wt% addition, the mean CO formation rates for ZIF-8, b-GQDs/ZIF-8, y-GQDs/ZIF-8, and r-GQDs/ZIF-8 were 8.090 ± 0.286, 14.150 ± 0.400, 18.900 ± 0.422, and 23.507 ± 0.477 μmol·g^−1^·h^−1^, respectively; the corresponding CH_4_ rates were 0.900 ± 0.075, 1.860 ± 0.092, 2.863 ± 0.127, and 4.080 ± 0.148 μmol·g^−1^·h^−1^ (mean ± SD, *n* = 3; Figure 5c and Table S5). Relative to pristine ZIF-8, nominal 4 wt% r-GQDs/ZIF-8 showed approximately 2.9- and 4.5-fold higher mean CO and CH_4_ rates. Its combined mean rate was 27.587 μmol·g^−1^·h^−1^. These differences are interpreted descriptively because no inferential statistical comparison was performed.

The activity order did not follow the fluorescence quantum-yield order of the isolated GQDs. This comparison shows that radiative emission efficiency alone is not a sufficient descriptor of composite performance. The extended optical response of r-GQDs, the XPS-observed surface-composition differences, and the EIS, photocurrent, and time-resolved PL trends are mutually consistent with regulator-dependent relaxation and transport behavior. Nevertheless, the present measurements do not separate these contributions from differences in surface basicity, actual GQD loading, CO_2_ adsorption, or other interfacial effects [30,31,37,39]. To position the present results in the broader field, Table 1 compares representative ZIF-8-based systems. The table deliberately reports key conditions because apparent rates depend strongly on irradiation, reactor mode, catalyst mass, additives, and gas composition. The nominal 4 wt% r-GQDs/ZIF-8 rate is similar in magnitude to the reported TiO_2_/ZIF-8 gas–solid system, lower than the TpPa/ZIF-8 COF hybrid, and far below the single-Ni-atom hierarchical ZIF-8 system; these more complex systems differ in active sites, architecture, and reaction conditions and are therefore not direct performance benchmarks.

Three independent five-cycle activity tests were performed for nominal 4 wt% r-GQDs/ZIF-8 (Figure 5d and Table S6). The mean combined CO and CH_4_ rate decreased from 27.537 ± 0.302 μmol·g^−1^·h^−1^ in the first cycle to 23.070 ± 0.295 μmol·g^−1^·h^−1^ in the fifth cycle, corresponding to 83.78 ± 0.54% retention. The results demonstrate measurable short-term reusability but also reveal progressive activity loss. Because post-reaction structural characterization was not available, the decay cannot be assigned uniquely to GQD loss, surface fouling, framework change, or experimental handling.

### 3.6. Surface-Chemical Comparison of ZIF-8 and r-GQDs/ZIF-8

**Figure 6:**
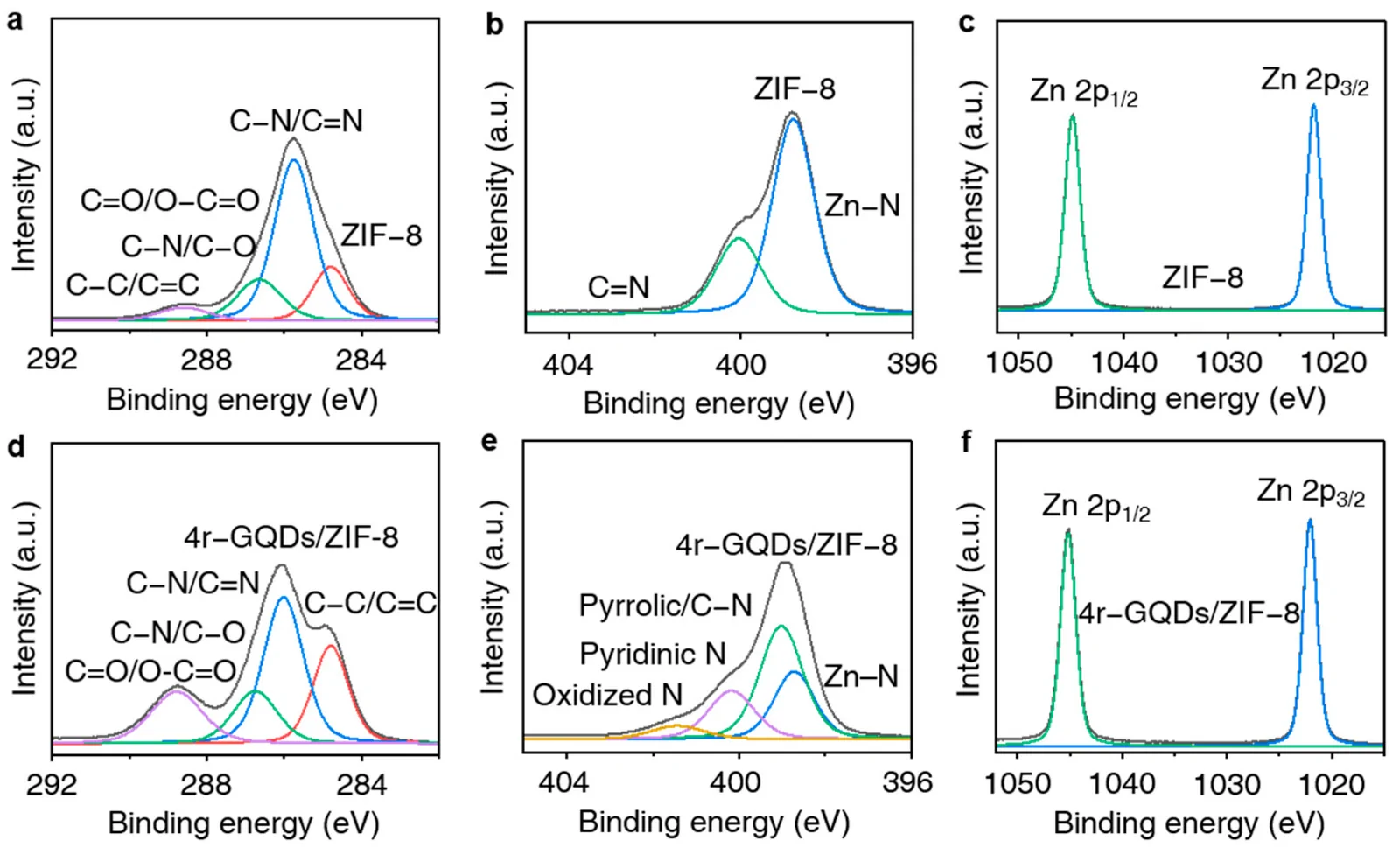
High-resolution XPS spectra of pristine ZIF-8 and nominal 4 wt% r-GQDs/ZIF-8. Panels (**a**–**c**) show the C 1s, N 1s, and Zn 2p regions of pristine ZIF-8, respectively, and panels (**d**–**f**) show the corresponding regions of 4 wt% r-GQDs/ZIF-8. The experimental spectra, fitted envelopes, and deconvoluted components are shown as grey/black, red, and colored curves, respectively. The fitted components are assigned to the bonding states labelled in each panel, including C–C/C=C, C–N/C=N, C–N/C–O, C=O/O–C=O, pyridinic N, pyrrolic/C–N, oxidized N, Zn–N, Zn 2p3/2, and Zn 2p1/2. Binding energies were referenced to the C 1s peak at 284.8 eV. The fitted component positions and surface compositions are summarized in Table S3.

High-resolution XPS spectra were used to compare pristine ZIF-8 and nominal 4 wt% r-GQDs/ZIF-8. Figure 6a–c shows the C 1s, N 1s, and Zn 2p regions of ZIF-8, while Figure 6d–f shows the corresponding regions of the composite. The measured surface composition changed from C/N/O/Zn = 54.2/27.8/2.1/15.9 at.% for ZIF-8 to 57.0/25.6/5.6/11.8 at.% for r-GQDs/ZIF-8 (Table S3).

The principal N 1s component shifted from 398.78 to 399.02 eV, while Zn 2p_3/2_ shifted from 1021.80 to 1022.06 eV (Figure 6b,c,e,f and Table S3). These modest differences are consistent with a changed surface chemical environment after composite preparation, but are not treated as direct evidence for a unique coordination bond, oxidation-state change, or electron-transfer direction.

### 3.7. Evidence Boundaries and Alternative Contributions

The combined measurements establish that regulator choice is associated with differences in surface composition, optical response, time-resolved PL decay, impedance, photocurrent, and photocatalytic activity. XPS provides direct surface-sensitive evidence for nitrogen-containing environments, while measurements on the composites show distinct relaxation and photoelectrochemical responses. However, the present dataset does not determine band positions, carrier-transfer direction, quantitative lifetimes, adsorbed CO_2_ intermediates, or a unique catalytic pathway.

The smaller Nyquist arc, higher transient photocurrent, and faster qualitative PL decay of r-GQDs/ZIF-8 are mutually consistent with a distinct photoinduced relaxation and transport response under the measurement conditions. These observations may contribute to the higher catalytic rate, but they neither identify the direction of electron flow nor exclude effects from surface composition, actual GQD loading, surface basicity, or CO_2_ adsorption. Likewise, the modest XPS shifts and FT-IR/Raman changes indicate altered chemical environments but do not establish a unique interfacial bond or catalytic adsorption site.

The inverse ordering of isolated-GQD fluorescence quantum yield and composite photocatalytic activity remains an empirical correlation. The additional composite time-resolved PL data demonstrate that the relaxation dynamics also vary across the composite series; nevertheless, the decays were not converted into model-dependent lifetimes. The combined optical and photoelectrochemical evidence therefore supports regulator-dependent behavior but not a single productive electron-transfer route [24,25,26,27,30,31].

The broad activity maximum at nominal additions of 3–5 wt% may reflect competing effects of GQD retention, aggregation, light absorption, interfacial contact, surface accessibility, and reactant transport [37,38]. In the absence of measured final GQD contents, Brunauer–Emmett–Teller (BET) surface-area and pore-volume data, and CO_2_-adsorption or activation measurements, pore blocking and CO_2_ enrichment are not claimed. Dark, catalyst-free, and N_2_ controls produced signals below 0.8% of those measured under CO_2_, light, and catalyst conditions (Table S8), supporting the dependence of the observed activity on these three factors. However, isotopic carbon-source verification was not available, and the oxidation half-reaction and possible H_2_/O_2_ products were not quantified. The reported rates are therefore treated as comparative measurements under the stated conditions rather than a complete reaction balance or definitive isotope-resolved carbon-source assignment. Future verification should employ ^13^CO_2_ coupled with gas chromatography–mass spectrometry (GC-MS) (or an equivalent isotope-resolved method) to confirm the carbon source of CO and CH_4_; because such measurements were not performed in the present study, no isotope-based origin claim is made.

## 4. Conclusions

GQD samples were prepared from pyrene using urea, melamine, and 2,4-pyridinedicarboxylic acid as nitrogen-containing regulators and were combined with ZIF-8 for visible-light-driven CO_2_ reduction. FT-IR, Raman, XRD, representative high-magnification TEM, XPS, and optical measurements showed regulator-dependent structural, surface-compositional, and optical differences, while the principal ZIF-8 diffraction peaks were retained after composite preparation. Among the tested samples, nominal 4 wt% r-GQDs/ZIF-8 gave the highest observed mean CO and CH_4_ formation rates of 23.51 ± 0.48 and 4.08 ± 0.15 μmol·g^−1^·h^−1^, corresponding to approximately 2.9- and 4.5-fold increases over pristine ZIF-8. This sample also showed the lowest fitted R_ct_, the highest mean photocurrent density, and the fastest qualitative time-resolved PL decay among the compared composites. Across three independent five-cycle tests, 83.78 ± 0.54% of the initial combined rate was retained. The results support correlations among regulator identity, surface composition, optical relaxation, photoelectrochemical response, and catalytic activity; they do not establish a unique interfacial charge-transfer mechanism. The absence of quantitative GQD loading, adsorption/textural characterization, isotope-resolved carbon-source verification, a complete product balance, quantitative time-resolved photoluminescence (TRPL) parameters, and post-cycle structural analysis remains an important limitation. The activity is therefore positioned as a measurable improvement over the matched pristine-ZIF-8 control under the present aqueous visible-light protocol, rather than as a state-of-the-art or directly normalized performance record.

## Figures and Tables

**Table 1:** Contextual comparison with selected ZIF-8-based CO_2_ photoreduction reports. The entries are not a normalized performance ranking because the light source, reactor configuration, catalyst mass, and reaction medium differ substantially.

Photocatalyst	Reported Rate (μmol·g^−1^·h^−1^)	Key Reaction Conditions	Interpretive Context
Pristine ZIF-8 (this work)	CO 8.09; CH_4_ 0.90	300 W Xe lamp, λ > 420 nm; 10 mg catalyst in 10 mL water; CO_2_-purged sealed reactor.	Within-study baseline; *n* = 3.
Nominal 4 wt% r-GQDs/ZIF-8 (this work)	CO 23.51; CH_4_ 4.08	Same conditions as the ZIF-8 baseline.	Nominal loading only; *n* = 3.
TiO_2_/ZIF-8-G2 [40]	CO 21.74	40 W LED; gas–solid mode; 1 mg catalyst and H_2_O vapor; no photosensitizer or sacrificial agent.	Different reactor and phase; comparable only as context.
TpPa/ZIF-8 [41]	CO 43.94 (pure CO_2_); 84.87 (10% CO_2_)	40 W LED; gas–solid mode; H_2_O was used as the reductant.	COF/ZIF-8 hybrid under different gas conditions.
Ni single-atom hierarchical ZIF-8 [4]	CO 4200	300 W Xe lamp, λ > 400 nm; Ni active sites and ordered hierarchical pores.	A substantially more complex active-site/porosity design; not a like-for-like benchmark.

## Data Availability

The raw data supporting the conclusions of this article will be made available by the authors on request.

## Supplementary Materials

The following supporting information can be downloaded at: https://www.mdpi.com/article/10.3390/nano16181126/s1, Figure S1: Supplementary structural and spectroscopic characterization: (a) XPS survey spectra of b-GQDs, y-GQDs, and r-GQDs; (b) FT-IR spectra and (c) XRD patterns of pristine ZIF-8 and the nominal 4 wt% b-GQDs/ZIF-8, y-GQDs/ZIF-8, and r-GQDs/ZIF-8 composites. The FT-IR and XRD comparisons are interpreted qualitatively and are not used alone to establish a specific interfacial bond or retained GQD loading; Table S1: Surface elemental composition of the regulator-derived GQD samples from XPS; Table S2: N 1s component positions and relative areas derived from the processed XPS fits; Table S3: Surface composition and selected XPS peak positions for ZIF-8 and nominal 4 wt% r-GQDs/ZIF-8; Table S4: Relative fluorescence quantum yields of the isolated regulator-derived GQD samples; Table S5: Photocatalytic CO and CH_4_ formation rates for pristine ZIF-8 and the nominal GQD loading series. Formation rates are reported in μmol g^−1^ h^−1^ as mean ± SD from three independent photocatalytic runs (*n* = 3); Table S6: Combined CO and CH_4_ formation rates and retention during three independent five-cycle tests of nominal 4 wt% r-GQDs/ZIF-8. Values are mean ± SD (*n* = 3); Table S7: Principal FT-IR band positions and qualitative spectral differences of the GQD samples, pristine ZIF-8, and nominal 4 wt% GQDs/ZIF-8 composites. Values are approximate band positions obtained from the original spectra. Within the grouped entries, the values are listed in the order b-, y-, and r-derived samples. Because the spectra were not subjected to peak fitting or integrated-area analysis, intensity differences and small shifts are interpreted qualitatively; Table S8: Control experiments for nominal 4 wt% r-GQDs/ZIF-8. Formation rates are reported in μmol g^−1^ h^−1^ as mean ± SD from three independent runs (*n* = 3); Table S9: Equivalent-circuit parameters obtained from EIS fits of three independently prepared electrodes per sample using the R_s_-(Rct||CPE) model; Table S10: Summary of fitted charge-transfer resistance and photocurrent density. Values are mean ± SD from three independently prepared electrodes (*n* = 3).
