# Supplementary material for: Effect of Nitrogen-Containing Regulators on Graphene Quantum Dot/ZIF-8 Composites for Photocatalytic CO2 Reduction
Source: Nanomaterials (Basel). 2026 Sep 8;16(18):1126. doi: 10.3390/nano16181126 (PMC13610354; doi:10.3390/nano16181126)
Supplement: Supplementary file 1 [file nanomaterials-16-01126-s001.zip › nanomaterials-4556784-supplementary.pdf]

Supplementary Information

Effect of Nitrogen-Containing Regulators on Graphene Quantum Dot/ZIF-8 Composites for Photocatalytic CO_2 Reduction

Lei Wang, Xinyuan Gao, Shang Li, Shuangyan Li and Weitao Li*

College of Materials Electronics and Energy Storage, Zhongyuan University of Technology, Zhengzhou 450007, China

Supplementary figures

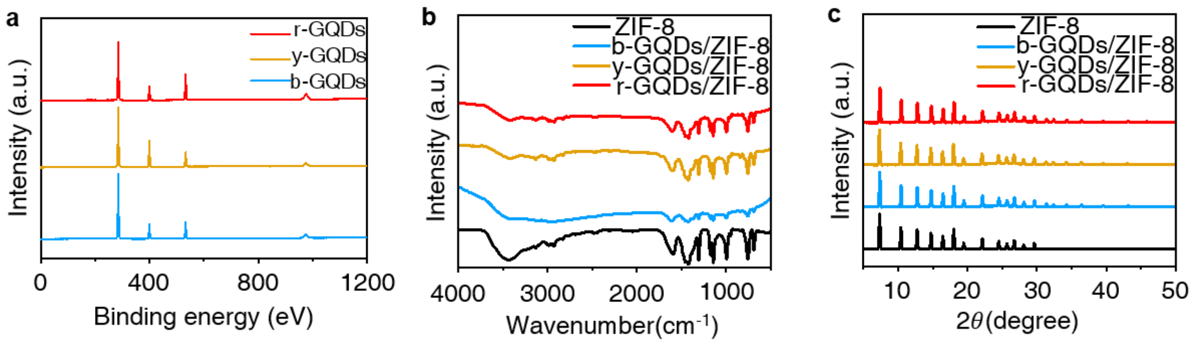

Figure S1. Supplementary structural and spectroscopic characterization: (a) XPS survey spectra of b-GQDs, y-GQDs, and r-GQDs; (b) FT-IR spectra and (c) XRD patterns of pristine ZIF-8 and the nominal 4 wt% b-GQDs/ZIF-8, y-GQDs/ZIF-8, and r-GQDs/ZIF-8 composites. The FT-IR and XRD comparisons are interpreted qualitatively and are not used alone to establish a specific interfacial bond or retained GQD loading.

Supplementary tables

Table S1. Surface elemental composition of the regulator-derived GQD samples from XPS.

Sample	C (at.%)	N (at.%)	O (at.%)
b-GQDs	75.6	9.2	15.2
y-GQDs	69.8	16.4	13.8
r-GQDs	67.4	8.7	23.9

Table S2. N 1s component positions and relative areas derived from the processed XPS fits.

Sample	Pyridinic N	Pyrrolic/amine N	Graphitic N	Oxidized N
b-GQDs	398.45 eV; 24.0%	399.85 eV; 44.0%	401.15 eV; 25.5%	402.55 eV; 6.5%
y-GQDs	398.52 eV; 31.0%	399.92 eV; 46.0%	401.18 eV; 18.5%	402.55 eV; 4.5%
r-GQDs	398.50 eV; 43.5%	399.95 eV; 31.5%	401.22 eV; 19.0%	402.60 eV; 6.0%

Table S3. Surface composition and selected XPS peak positions for ZIF-8 and nominal 4 wt% r-GQDs/ZIF-8.

Sample	C/N/O/Zn (at.%)	Principal N 1s (eV)	Zn $2p_{3/2}$ (eV)	Zn $2p_{1/2}$ (eV)
ZIF-8	54.2/27.8/2.1/15.9	398.78	1021.80	1044.85
4 wt% r-GQDs/ZIF-8	57.0/25.6/5.6/11.8	399.02	1022.06	1045.15

Table S4. Relative fluorescence quantum yields of the isolated regulator-derived GQD samples.

Sample	Maximum relative quantum yield (%)
b-GQDs	38.35
y-GQDs	32.13
r-GQDs	20.09

Table S5. Photocatalytic CO and CH_4 formation rates for pristine ZIF-8 and the nominal GQD loading series. Formation rates are reported in $\mu mol \cdot g^{-1} \cdot h^{-1}$ as mean ± SD from three independent photocatalytic runs (n = 3).

Sample	CO-1	CO-2	CO-3	CO mean ± SD	CH_4-1	CH_4-2	CH_4-3	CH_4 mean ± SD
ZIF-8	8.36	7.79	8.12	8.090 ± 0.286	0.97	0.82	0.91	0.900 ± 0.075
1 wt% b-GQDs/ZIF-8	10.62	9.98	10.45	10.350 ± 0.332	1.31	1.14	1.24	1.230 ± 0.085
2 wt% b-GQDs/ZIF-8	12.41	11.73	12.27	12.137 ± 0.359	1.39	1.58	1.46	1.477 ± 0.096
3 wt% b-GQDs/ZIF-8	14.02	14.67	14.15	14.280 ± 0.344	1.83	1.66	1.79	1.760 ± 0.089
4 wt% b-GQDs/ZIF-8	14.51	13.72	14.22	14.150 ± 0.400	1.78	1.96	1.84	1.860 ± 0.092
5 wt% b-GQDs/ZIF-8	13.44	14.14	13.79	13.790 ± 0.350	1.72	1.86	1.68	1.753 ± 0.095
1 wt% y-GQDs/ZIF-8	12.82	12.17	12.56	12.517 ± 0.327	1.72	1.51	1.64	1.623 ± 0.106
2 wt% y-GQDs/ZIF-8	15.11	15.82	15.39	15.440 ± 0.358	1.96	2.15	2.02	2.043 ± 0.097
3 wt% y-GQDs/ZIF-8	17.46	16.71	17.29	17.153 ± 0.393	2.35	2.57	2.41	2.443 ± 0.114
4 wt% y-GQDs/ZIF-8	19.21	18.42	19.07	18.900 ± 0.422	2.96	2.72	2.91	2.863 ± 0.127
5 wt% y-GQDs/ZIF-8	18.31	19.16	18.74	18.737 ± 0.425	2.79	3.06	2.92	2.923 ± 0.135
1 wt% r-GQDs/ZIF-8	15.34	14.61	15.08	15.010 ± 0.370	2.20	2.01	2.16	2.123 ± 0.100
2 wt% r-GQDs/ZIF-8	18.22	18.91	18.47	18.533 ± 0.349	2.66	2.85	2.74	2.750 ± 0.095
3 wt% r-GQDs/ZIF-8	21.58	20.72	21.29	21.197 ± 0.438	3.34	3.57	3.42	3.443 ± 0.117
4 wt% r-GQDs/ZIF-8	23.91	22.98	23.63	23.507 ± 0.477	4.18	3.91	4.15	4.080 ± 0.148
5 wt% r-GQDs/ZIF-8	22.61	23.47	23.06	23.047 ± 0.430	3.82	4.12	3.96	3.967 ± 0.150

Table S6. Combined CO and CH_4 formation rates and retention during three independent five-cycle tests of nominal 4 wt% r-GQDs/ZIF-8. Values are mean ± SD (n = 3).

Cycle	Run 1	Run 2	Run 3	Combined rate, mean ± SD ($\mu mol \cdot g^{-1} \cdot h^{-1}$)	Retention, mean ± SD (%)
1	27.31	27.88	27.42	27.537 ± 0.302	100.00 ± 0.00
2	26.18	26.59	26.05	26.273 ± 0.282	95.41 ± 0.43
3	25.31	25.74	25.09	25.380 ± 0.331	92.17 ± 0.60
4	24.10	24.46	23.92	24.160 ± 0.275	87.74 ± 0.51
5	23.01	23.39	22.81	23.070 ± 0.295	83.78 ± 0.54

Retention was calculated separately for each independent run relative to its combined CO and CH_4 formation rate in cycle 1, which was defined as 100%; the three retention values were then summarized as mean ± SD.

Table S7. Principal FT-IR band positions and qualitative spectral differences of the GQD samples, pristine ZIF-8, and nominal 4 wt% GQDs/ZIF-8 composites. Values are approximate band positions obtained from the original spectra. Within the grouped entries, the values are listed in the order b-, y-, and r-derived samples. Because the spectra were not subjected to peak fitting or integrated-area analysis, intensity differences and small shifts are interpreted qualitatively.

Spectral region (cm^{-1})	Isolated GQDs: b/y/r	Pristine ZIF-8	Nominal 4 wt% composites: b/y/r	Tentative assignment	Qualitative comparison
3700–3000	3421/3388/3351	3441	3390/3421/3418	O–H/N–H stretching	Broad-band positions and profiles varied among the regulator-derived samples; no quantitative intensity ranking was assigned.
1650–1500	1587/1612/1595	1597	1610/1601/1603	C=C, C=N, and ring stretching	The composites showed only small position differences relative to pristine ZIF-8; these shifts do not establish a new interfacial bond.
1480–1350	1394/1417/1374	1427	1425/1427/1429	Ring vibration and C–N-related modes	The composite bands remained close to the characteristic ZIF-8 region.
1330–1050	1269, 1181/1226, 1142/1281, 1123	1308, 1144	1308, 1144/1308, 1146/1310, 1146	C–N/C–O-related vibrations and imidazolate fingerprint modes	Modest profile and intensity differences were observed, but integrated intensities were not determined.
850–680	780/838, 762/842	758, 688	760, 694/758, 690/760, 690	Ring out-of-plane deformation	The characteristic ZIF-8 fingerprint bands were retained after composite preparation.
500–400	—	421	423/423/423	Zn–N stretching	Retention of the low-wavenumber Zn–N band is consistent with preservation of the ZIF-8 coordination framework.

Table S8. Control experiments for nominal 4 wt% r-GQDs/ZIF-8. Formation rates are reported in $\mu mol \cdot g^{-1} \cdot h^{-1}$ as mean ± SD from three independent runs (n = 3).

Condition	CO-1	CO-2	CO-3	CO mean ± SD	CH_4-1	CH_4-2	CH_4-3	CH_4 mean ± SD
CO_2 + light + catalyst	23.91	22.98	23.63	23.507 ± 0.477	4.18	3.91	4.15	4.080 ± 0.148
CO_2 + dark + catalyst	0.18	0.11	0.15	0.147 ± 0.035	0.04	0.02	0.03	0.030 ± 0.010
CO_2 + light, no catalyst	0.09	0.13	0.08	0.100 ± 0.026	0.02	0.03	0.02	0.023 ± 0.006
N_2 + light + catalyst	0.12	0.16	0.10	0.127 ± 0.031	0.03	0.02	0.03	0.027 ± 0.006

Table S9. Equivalent-circuit parameters obtained from EIS fits of three independently prepared electrodes per sample using the R_s-(Rct||CPE) model.

Sample	Electrode	R_s (Ω)	Rct (Ω)	CPE-Q	CPE-n	χ^2
ZIF-8	1	18.7	812	7.8×10^{-5}	0.82	1.7×10^{-3}
ZIF-8	2	19.4	775	8.2×10^{-5}	0.80	2.1×10^{-3}
ZIF-8	3	18.9	836	7.5×10^{-5}	0.83	1.9×10^{-3}
4 wt% b-GQDs/ZIF-8	1	18.1	545	9.6×10^{-5}	0.84	1.5×10^{-3}
4 wt% b-GQDs/ZIF-8	2	18.8	522	1.02×10^{-4}	0.83	1.8×10^{-3}
4 wt% b-GQDs/ZIF-8	3	18.4	561	9.3×10^{-5}	0.85	1.6×10^{-3}
4 wt% y-GQDs/ZIF-8	1	17.9	371	1.18×10^{-4}	0.86	1.4×10^{-3}
4 wt% y-GQDs/ZIF-8	2	18.2	352	1.24×10^{-4}	0.85	1.5×10^{-3}
4 wt% y-GQDs/ZIF-8	3	17.7	386	1.15×10^{-4}	0.87	1.3×10^{-3}
4 wt% r-GQDs/ZIF-8	1	17.5	238	1.42×10^{-4}	0.89	1.2×10^{-3}
4 wt% r-GQDs/ZIF-8	2	17.9	221	1.48×10^{-4}	0.88	1.4×10^{-3}
4 wt% r-GQDs/ZIF-8	3	17.6	246	1.39×10^{-4}	0.90	1.1×10^{-3}

Table S10. Summary of fitted charge-transfer resistance and photocurrent density. Values are mean ± SD from three independently prepared electrodes (n = 3).

Sample	Rct (Ω), mean ± SD	Photocurrent density ($\mu A \cdot cm^{-2}$), mean ± SD
ZIF-8	807.7 ± 30.7	1.847 ± 0.070
4 wt% b-GQDs/ZIF-8	542.7 ± 19.6	3.440 ± 0.098
4 wt% y-GQDs/ZIF-8	369.7 ± 17.0	4.750 ± 0.118
4 wt% r-GQDs/ZIF-8	235.0 ± 12.8	6.333 ± 0.127

Data interpretation note. Photocatalytic activity, cycling, control, photocurrent, and EIS summary values are reported as mean ± SD from three independent runs or independently prepared electrodes, as specified in the corresponding captions. The absolute DC potential was not retained in the exported electrochemical acquisition table; therefore, the EIS and photocurrent results are used only for within-series comparison. XPS, FT-IR, and time-resolved PL interpretations remain qualitative where replicate-level fitting or quantitative model validation was not available.
